# Galactose treatment of a PGM1 patient presenting with restrictive cardiomyopathy

**DOI:** 10.1002/jmd2.12177

**Published:** 2020-10-19

**Authors:** Sarah E. Donoghue, Susan M. White, Tiong Yang Tan, Remi Kowalski, Eva Morava, Joy Yaplito‐Lee

**Affiliations:** ^1^ Department of Metabolic Medicine Royal Children's Hospital Melbourne Victoria Australia; ^2^ Victorian Clinical Genetics Services Murdoch Children's Research Institute Melbourne Victoria Australia; ^3^ Department of Paediatrics University of Melbourne Melbourne Victoria Australia; ^4^ Department of Cardiology Royal Children's Hospital Melbourne Victoria Australia; ^5^ Department of Clinical Genomics Mayo Clinic Rochester Minnesota USA; ^6^ Department of Pediatrics University Hospitals Leuven Leuven Belgium

**Keywords:** galactose, PGM1‐CDG, restrictive cardiomyopathy

## Abstract

We report a patient diagnosed with PGM1‐CDG at 11 years of age after two biallelic likely pathogenic variants in *PGM1* were found on research genomic sequencing. To our knowledge, he is the first patient with PGM1‐CDG to be reported with a restrictive cardiomyopathy. Other clinical manifestations included cleft palate, asymptomatic elevated transaminases, intellectual disability and myopathy resulting in exercise intolerance. He was trialed on oral galactose therapy in increasing doses for 18 weeks to assess if there was any biochemical and clinical benefit. His galactose was continued for a further 9 months beyond the initial galactose treatment period due to improvements in exercise tolerance and myopathy. Treatment with galactose demonstrated an improvement in liver function and myopathy with improved exercise tolerance. Treatment with galactose for 15 months did not change heart function and exercise stress test results were stable.

**Synopsis**

Presentation with a restrictive cardiomyopathy may be a clinical feature of PGM1‐CDG.

## 1 INTRODUCTION

Congenital disorders of glycosylation (CDG) are a group of genetic metabolic conditions that alter protein glycosylation.^1^ Phosphoglucomutase deficiency (PGM1‐CDG) has important roles in glycogenolysis and glyconeogenesis as it catalyzes the bidirectional conversion of glucose 1‐phosphate and glucose 6‐phophate.^1^, ^2^, ^3^ In addition to the maintenance of glucose homeostasis, PGM1‐CDG has a role in protein N‐linked glycosylation, which is very important in post‐translational modification.^4^ The defects in glycosylation affect transport proteins, coagulation factors and organ system development.^4^, ^5^ Previously reported features of PGM1‐CDG included hepatopathy, hypoglycemia, congenital craniofacial malformations, myopathy, coagulopathy, endocrine deficiencies, dilated cardiomyopathy, strabismus, seizures and intellectual disability.^6^

Oral d‐galactose supplementation has been trialed in patients with PGM1 deficiency, which demonstrated beneficial effects in glycosylation with both transferrin isoelectric focusing and in vitro studies examining fibroblasts.^2^, ^7^ Other reported benefits from this trial included normalization or improvement of abnormal coagulation and liver function abnormalities.^7^ It has also been demonstrated that d‐galactose supplementation is safe when given orally in doses of 1.5 g/kg/day up to a maximum dose of 50 g daily.^7^

## 2 CASE REPORT

Our patient is the second child of three children born to non‐consanguineous Caucasian parents. During pregnancy, an increased nuchal translucency was detected of greater than 6 mm and short long bones were identified on a first trimester ultrasound performed at 13 weeks of gestation. He was born at 36 weeks of gestation via normal vaginal delivery, with birth weight 3 kg (17.76th centile, Z = −0.92), length 46 cm (6.86th centile, Z = −1.49) and head circumference 33.6 cm (14.33th centile, Z = −1.07). There is a family history of learning difficulties affecting his parents, brother and extended family members.

He was diagnosed with a midline cleft palate postnatally and required readmission due to feeding difficulties. Distinctive features were noted including a round face, depressed nasal bridge, simple morphology to the ears and thin eyebrows, features which were shared by his mother. His midface was noted to be relatively hypoplastic compared to his family members. He had bilateral single palmar creases and short fifth fingers. Further investigations at the time revealed an atrial septal defect measuring 8 mm. A skeletal survey at 1 month of age showed mild epiphyseal delay and slight rounding of vertebral bodies.

There were growth concerns in the first 12 months of life and a gastrostomy was inserted at 14 months of age because of failure to thrive. This was removed at 18 months following palate repair and an improvement in growth. His weight crossed centiles and regained to the 5th centile, however his length remained on the 1st centile until 10 years of age when his growth velocity improved. At 12 years of age, he reached the 17th centile for height and weight increased to the 60th centile.

A restrictive cardiomyopathy was diagnosed at 5 years of age after previous earlier follow‐up echocardiograms demonstrated no cardiomyopathy. Serial echocardiography demonstrated normal left and right ventricular function in the context of a dilated left atrium. Cardiac magnetic resonance imaging (MRI) was performed at age 11 years demonstrating no pericardial involvement. His echocardiogram demonstrated a mild reduction in systolic function after the age of 11 years and he developed features of a combined restrictive and dilated cardiomyopathy (DCM) with predominance of restrictive form in the next 6 months. The evolution of the echocardiogram findings is summarized in Table 1.

**TABLE 1:** Evolution of initial echocardiogram findings in patient with PGM1‐CDG

Age					
Echocardiogram	3.5 years	5.5 years	6 years	8 years	10 years
LVEDd (cm)	3.4	4.0	4.5	4.6	4.7
Normal range	2.3 to 3.4	2.9 to 3.9	2.9 to 4.0	3.1 to 4.2	3.4 to 4.7
Z‐score	1.9	2.7	3.8	3.5	2.0
Fractional shortening (%)	32.2	33.6	31.1	26.7	33.8
EF (%)	61.6	65.2	59	51.3	54.1
Septal thickness (cm)	0.49	0.56	0.40	0.7	0.67
Normal range	0.25 to 0.67	0.33 to 0.7	0.26 to 0.79	0.35 to 0.75	0.35 to 0.79
Z‐score	0.28	0.51	−0.93	1.5	0.33
Atrial dilation	Normal size	Normal RA size	Mild RA dilation		Normal RA size
		Severe LA dilation	Severe LA dilation	Severe LA dilatation with septal bowing	Severe LA dilatation with rightward septal bowing
RUV A wave reversal velocity (cm/s)	—	78.2 cm/s	—	—	66
MV E/A ratio		3.0	1.5	‐	2.2
Tissue Doppler velocities	Normal	Normal	Normal	Normal	Normal

Abbreviations: EF, ejection fraction; LA, left atrium; LVEDd, left ventricular end diastolic dimension; RA, right atrium; RUPV, right upper pulmonary vein.

Brain MRI at age 10 years was normal. Our patient had a normal banded karyotype and FISH testing for 22q11.21 in the neonatal period. Microarray showed a maternally inherited 0.2 Mb duplication of chromosome 5q35.5 that was novel. The duplication contained *PROP1*, in which biallelic mutations have been associated with hypopituitarism, and three other genes whose function is unknown. The significance of this duplication is unknown. A cardiomyopathy gene panel encompassing 63 cardiomyopathy genes did not identify any pathogenic variants.

Further research genomic testing by a custom‐designed Agilent SureSelect craniofacial panel comprising 79 genes as described previously by Tan et al^8^ at age 11 years identified a previously reported variant in the *PGM1* gene, c.1042 G>C, p.(Gly348Arg) and a novel variant c. 1051 C>T, p.(Arg351Trp). Visual inspection of sequencing reads on IGV confirmed that the variants were in trans. Both variants are present in heterozygous state in low frequencies in the gnomAD population database, and have not been reported in affected individuals. Both variants affected residues located within the PGM_PMM_III functional domain of the PGM1 protein and both amino acid residues are highly conserved. *In silico* software predictions that the variants were both disease causing. Using the American College of Medical Genetics for assessing pathogenicity described by Richards et al^9^ both variants were classified as likely pathogenic. Serum transferrin isoforms performed at the age of 11 years old were abnormal with elevated disialotransferrin and decreased tetrasialotransferrin.

The patient was started on d‐galactose age 12 years with increasing dosage as per Table 1. The galactose supplementation was continued beyond week 18 for a period of 9 months. One examiner (SD) performed clinical examination and scoring according to the Tulane PGM1‐CDG Rating Scale (TPCRS) that was previously used by Wong et al^6^ to define phenotype and severity. Clinical evaluation, laboratory studies and assessments were followed as per Table 1.

## 3 RESULTS

Galactose supplementation was well tolerated with no reportable adverse events. Serial measures with the TPCRS demonstrated an improvement in the score. This was mainly due to improvement in myopathy and hearing where he no longer required hearing aids due to improvements on serial audiology assessments. During the period of galactose supplementation, our patient reported improved exercise tolerance, a reduction in muscle pain and improved quality of life as he was now able to run and participate in low impact sporting activities and walking. His muscle strength also significantly improved on clinical assessment. Creatine kinase (CK) levels fluctuated through the study and did not correlate to clinical symptoms (Table 2).

**TABLE 2:** Evolution of clinical, pathology, imaging and functional tests in a PGM1‐CDG patient while on galactose supplementation

Parameter	Baseline	6 wk	12 wk	18 wk	3 mo maintenance^a^	6 mo maintenance	9 mo maintenance
Galactose dose	—	0.5 g/kg/d	1 g/kg/d	50 g daily	50 g daily	50 g daily	50 g daily
Clinical scoring							
Tulane PGM1‐CDG rating scale							
Total score	12	10	10	8	9	9	8
I. Current function							
Vision	0	0	0	0	0	0	0
Hearing	1	1	1	0	0	0	0
Communication	0	0	0	0	0	0	0
Feeding	0	0	0	0	0	0	0
Mobility	0	0	0	0	0	0	0
II. System specific involvement							
Seizures	0	0	0	0	0	0	0
Encephalopathy	0	0	0	0	0	0	0
Haemostatic	1	0	1	1	1	1	1
Gastrointestinal	1	0	0	0	0	0	0
Endocrine	0	0	0	0	0	0	0
Respiratory	0	0	0	0	0	0	0
Cardiac	2	2	2	2	2	2	2
Renal	0	0	0	0	0	0	0
Liver	1	1	1	1	1	1	1
Blood	0	1	1	1	1	0	0
Congenital malformation	3	3	3	3	3	3	3
III. Current clinical assessment							
Growth	0	0	0	0	0	0	0
Vision with glasses	0	0	0	0	0	0	0
Strabismus, abnormal eye movement	1	1	1	1	1	1	1
Myopathy	2	1	0	0	0	0	0
Ataxia	0	0	0	0	0	0	0
Pyramidal	0	0	0	0	0	0	0
Extrapyramidal	0	0	0	0	0	0	0
Neuropathy	0	0	0	0	0	0	0
Weight kg (centile)^b^	41.8 (58th)	43.3 (62nd)	43.4 (60th)	44.7 (63rd)	45.5 (62nd)	48 (66th)	50 (68th)
Height cm (centile)	140 (12th)	142.1 (17th)	142.1 (14th)	143 (15th)	144.4 (15th)	146.8 (17th)	148.3 (17th)
Pathology							
Full blood count							
Hemoglobin (120‐160)	118	115	115	116	119	124	121
Platelets (150‐400)	179	161	183	214	164	208	171
White cells	Normal	Normal	Normal	Normal	Normal	Normal	Normal
Electrolytes, urea, creatinine	N	N	N	N	N	N	N
TSH (0.5‐4.5 mIU/L)	1.88	2.1	3.76	3.4	2.1	3.21	4.06
Coagulation testing							
INR (0.8‐1.2)	1.1	1.2	1.2	1.2	1.1	**1.4**	1.2
APTT (27‐44 s)	40	38	39	41	41	42	39
Fibrinogen (1.5‐4.3 g/L)	2.7	3.2	2.8	3.3	2.6	3.2	2.7
PT (11.5‐14.5 s)	—	—	—	—	—	—	14.7
Anti‐thrombin III (70%‐140%)	—	—	75	87	98	**55**	**69**
Factor XI (60%‐180%)	**55**	—	65	**56**	95	**29**	88
Factor IX (60%‐200%)	112	—	122	96	98	85	69
Liver function tests							
ALT (10‐35)	**95**	**61**	**45**	**47**	**63**	**46**	**57**
ALP (100‐350)	190	163	206	187	211	174	167
GGT (0‐40)	16	17	16	19	15	19	20
Lactate dehydrogenase (313‐618)	515	179	237	196	230	203	187
Creatine kinase (40‐240 U/L)	**258**	**306**	**1431**	**295**	**1002**	**838**	**303**
Lipid profile	N	N	N	N	N	N	N
Copper	N	N	N	N	N	N	N
Caeruloplasmin	N	N	N	N	N	N	N
IGF1 (16.52‐65.33 nmol/L)	19	29.6	31.1	39	19.7	31	25.7
IgFB3 (85.7‐211.9 nmol/L)	—	109.7	104.3	121.1	84.7	116	107
Serum transferrin isoforms							
Disialotransferrin (0‐3)	**14**	**5**	3	2.3	**12.8**	**5.7**	**5.3**
Trisialotransferrin (0‐8)	6	5.4	4.9	4.4	7.8	6.3	5.2
Tetrasialotransferrin (71‐84)	**68**	71	77	78	**64**	73	74
Pentasialotransferrin (10‐20)	12	19	15	16	15	15	15
Imaging and functional tests							
6‐min walk test (m)	415	447	428	490	460	465	499
Echocardiogram							
LVEDd (cm)	5.3			5.2			5.4
Normal range	3.6 to 5.0			3.7 to 5.1			3.8 to 5.2
Z‐score	2.9			2.2			2.5
Fractional shortening (%)	29.1			31.1			34.9
EF (%)	55.5			58.4			63.6
Septal thickness (cm)	0.48			0.74			0.66
Normal range	0.38 to 0.95			0.42 to 0.99			0.44 to 1.0
Z‐score	−1.3			0.29			−0.43
Atrial dilatation	Normal RA size			Normal RA size			Normal RA size
	Severe LA dilatation with rightward septal bowing			Severe LA dilatation with rightward septal bowing			Severely dilated LA, atrial septum bows into RA
RUV A wave reversal velocity (cm/s)	55.9			61.5			91
MV E/A ratio	2.0			3.0			2.1
Tissue Doppler velocities	Normal			Normal			Normal
Exercise stress test	6.3 min ceased due to fatigue						7 min modified Bruce protocol
Liver ultrasound		Borderline hepatomegaly 13.2 cm		Borderline hepatomegaly 13.2 cm			Normal liver ultrasound, no hepatomegaly

Abbreviations: LVEDd, left ventricular end diastolic dimension; EF, ejection fraction; RA, right atrium; LA, left atrium.

^a^ Non‐compliance with daily supplementation when reviewed.

^b^ Growth measured using—CDC Boys 2 to 20 year old growth charts.

The distance walked on the 6‐minute walk test (6MWT) was improved by 20% with consistent galactose supplementation. Three months following the initial 18‐week period of treatment, our patient was not always compliant with treatment and despite walking less distance on the 6MWT, he did not have any recurrence of myopathy or myalgia. Serial echocardiogram was stable throughout the study. Exercise treadmill testing demonstrated a small improvement.

Biochemical parameters such as full blood count, renal function, thyroid function and copper and caeruloplasmin remained stable. The liver function demonstrated an improvement in transaminases and abdominal ultrasound demonstrated mild hepatomegaly on imaging at baseline and 18 weeks of treatment. On treatment, our patient demonstrated emergence of mild abnormalities on his coagulation, but these did not manifest as clinical events. The serum transferrin isoforms initially normalized on treatment with oral galactose, with the tetrasialotranferrin normalizing during the first 6 weeks of treatment and the disialotransferrin normalizing in the first 12 weeks of treatment. When galactose therapy was not maintained at the 3 months maintenance dose of galactose the transferrin isoforms demonstrated a type 1 CDG pattern with an increase in disialotransferrin and a decreased tetrasialotransferrin level.

## 4 DISCUSSION

We report the effects of oral galactose supplementation in a 12‐year‐old patient with genetically confirmed PGM1 deficiency who was diagnosed at the age of 11 years by research genomic sequencing with abnormal serum transferrin isoelectric focussing confirming a type 1 CDG pattern.

Our patient was treated with oral galactose supplementation for 15 months without reporting any significant side‐effects. This safety information is consistent with other case studies and cohorts of patients who have been treated with oral d‐galactose supplementation.^7^, ^10^

Our patient had an improvement in his exercise tolerance and muscle pain on galactose therapy. This demonstrates a similar benefit to other patients where their functional data has been measured on galactose supplementation.^10^ Despite fluctuation in the CK levels, the patient remained asymptomatic without any reported myalgia, which is similar to what has been reported in previous studies.^7^, ^10^ Our patient is the first reported patient to present with an initial restrictive cardiomyopathy evolving subsequently into a combined restrictive and dilated cardiomyopathy. He had received serial echocardiograms from infancy to monitor closure of his atrial septal defect that did not demonstrate any features of cardiomyopathy prior to the first echocardiogram demonstrating a restrictive cardiomyopathy when he was 5 years old. DCM is the most common type of cardiac anomaly in PGM1, detected in 12 out of the 57 patients reported up till now with PGM1‐CDG.^4^, ^5^, ^7^, ^10^, ^11^, ^12^, ^13^, ^14^ The finding of the initial restrictive cardiomyopathy in our patient may demonstrate that there is a broader cardiac phenotype in patients with PGM1‐CDG. Our patient described that prior to galactose supplementation his exercise tolerance had been limited by myalgia, but following the supplementation it was limited by dyspnea. Galactose did not demonstrably improve the echocardiogram or ECG results in our patient. The improvement on the serial audiology screening cannot be easily explained and may be due to less episodes of otitis media during warmer weather.

Intellectual disability has been reported in other patients with PGM1‐CDG.^11^, ^15^, ^16^ Our patient was diagnosed with a mild intellectual disability, but as other family members have been diagnosed with an intellectual disability this may be multifactorial. The significance of the novel chromosome duplication is also unclear. Similarly, the dysmorphic features in our patient may be familial, as he shared a close resemblance to his mother. We consider it most likely that our patient's dysmorphic features and learning difficulties are familial traits, while his cleft palate, midface hypoplasia, atrial septal defect and cardiomyopathy are due to his PGM1‐CDG.

Our patient demonstrated normal levels of TSH, IGF1 and IGFB3 at the outset of the study and these remained essentially normal with treatment, meaning that we were unable to demonstrate the same improvements in glycosylation as seen in in previous patients on galactose supplementation.^7^ He also demonstrated improvements in his liver function, which is similar to other patients that have been previously reported.^7^ In contrast to some of the previously reported patients, our patient started with normal coagulation parameters (international normalised ratio [INR], activated partial thromboplastin time [APTT], fibrinogen and prothrombin time) and demonstrated mild derangement on longer term galactose supplementation.^7^ His baseline factor XI was low and the improvement of his levels was not always maintained even when he was compliant with supplementation. These coagulation abnormalities did not manifest in significant bleeding events, but this demonstrates that glycosylated proteins should be monitored and potentially still requires treatment in patients who remain on long‐term galactose supplementation. The difficulty in maintaining long‐term correction of glycosylation has previously been reported and it may reflect that higher doses of galactose may be required for longer term correction.^12^

## 5 CONCLUSION

Although dilated cardiomyopathy is well reported in PGM1‐CDG, we have reported the first case of a restrictive cardiomyopathy evolving into a combined restrictive and dilated cardiomyopathy. Treatment with galactose in PGM1‐CDG improves liver function, myopathy and exercise tolerance. Transferrin isoforms can normalize on supplementation with oral galactose. Galactose seems to be reasonably well tolerated; however, normal glycosylation may not necessarily be achieved on smaller doses of galactose and this means that glycosylated proteins may still require monitoring and treatment.

## CONFLICT OF INTEREST

Sarah Donoghue, Susan White, Tiong Tan, Remi Kowalski, Eva Morava and Joy Yaplito‐Lee declare that they have no conflict of interest.

## AUTHOR CONTRIBUTIONS

All authors contributed to manuscript preparation. Sarah E. Donoghue was involved in the clinical assessment of the patient and follow up of galactose therapy. Susan M. White and Tiong Yang Tan were involved in clinical assessment of patient for dysmorphology. Tiong Yang Tan was involved with the development of the genetic testing and interpretation of results. Remi Kowalski was involved with cardiac assessments. Eva Morava contributed to the conception of the work. Joy Yaplito‐Lee contributed to the initial assessment and conception of the work.

## INFORMED CONSENT

Written and informed consent was obtained from the family for the genetic testing and publication. There was institutional approval for the use of galactose. All procedures followed were in accordance with the Helsinki Declaration of 1975, as revised in 2000.
